# Co-variation between blood stream infections with *Candida* species versus *Pseudomonas aeruginosa, Staphylococcus aureus*, and other isolates among 60 ICU patient cohorts

**DOI:** 10.1093/mmy/myag023

**Published:** 2026-03-19

**Authors:** James C Hurley

**Affiliations:** Melbourne Medical School, University of Melbourne, Parkville, 3052, Australia; Ballarat Health Services, Grampians Health, Ballarat, 3350, Australia; Ballarat Clinical School, Deakin University, Ballarat, 3350, Australia

**Keywords:** blood stream infection, intensive care unit, observational cohort, mechanical ventilation, *Staphylococcus aureus*, *Enterococcal* species, *Pseudomonas aeruginosa*, *Acinetobacter* species, *Candida* species, Coagulase-negative Staphylococci

## Abstract

**Background:**

*Staphylococcus aureus, Pseudomonas aeruginosa, Enterococcal* species, Coagulase-negative Staphylococci, *Acinetobacter* species, and *Candida* species are common blood stream infection (BSI) isolates among intensive care unit (ICU) cohorts. That *Candida* species might interact with bacteria at mucosal surfaces to facilitate invasive infections prompts the question as to the degree of co-variance between *Candida* species versus bacteria among BSI isolates.

**Objectives:**

To estimate the co-variance between *Candida* species versus each of these five bacteria as BSI isolates among ICU patient cohorts.

**Methods:**

The literature was searched opportunistically for studies reporting ICU patient cohorts listing *Candida* species among BSI isolates. The associations of the BSI incidence proportion per 100 patients were converted to logits and modelled using ellipse plots.

**Results:**

The median overall BSI incidence proportion was 7.8% (interquartile range; 4.8%–11.6%). Among 60 cohorts (50 publications), correlation with the incidence proportion of Candidemia was apparent for *Acinetobacter* species (correlation coefficient = 0.69), *Staphylococcus aureus* (0.47), and *Pseudomonas aeruginosa* (0.4) but less so for Coagulase-negative Staphylococci (0.37) and *Enterococcal* species (0.32).

**Conclusions:**

There are various degrees of co-variance between the BSI incidence proportion amongst the five types of bacteria and *Candida* species among ICU cohorts.

## Introduction

Approximately 10% of patients requiring prolonged stay in the intensive care units (ICU) acquire blood stream infections (BSI), and *Staphylococcus aureus, Pseudomonas aeruginosa, Enterococcal* species, *Acinetobacter* species, and *Candida* species are common isolates.

There is increasing interest in mechanisms by which *Candida* species facilitate invasive infections and whether *Candida* species colonization enhances colonization susceptibility, wherein it facilitates invasive infections by bacterial pathogens, resulting in BSI along with other clinically important infections.^1–3^ This process, termed ‘microbial hitchhiking’,^4^ would have population-level implications, especially for ICU cohorts, which are at high risk of acquiring BSI during their stay in ICU.

Whether these *Candida* species-mediated interactions increase the risk for bacterial BSI among patients would be difficult to study. Any evidence that these interactions occurred among the colonizing flora would raise the potential for preventive interventions using anti-microbial or anti-fungal-based decontamination. Some studies of anti-microbial-based decontamination interventions have been either inconclusive or have paradoxically raised the risk of BSI through rebound colonization and spillover to concurrent patients.^5–10^

Here, the first objective is to visually display the co-variance between the incidence proportions of candidemia and BSI with various bacterial isolates among ICU patient cohorts appearing in published reports. Second, to estimate this co-variance as correlation co-efficients with comparison among the five bacterial types of interest. Third, to estimate the possible impacts of group-level factors on these observed correlations.

## Methods

Being an analysis of published work, ethics committee review of this study was not required.

### Data sources

The literature search used extends and updates a search as undertaken previously.^10–13^ In brief, an opportunistic search was undertaken within Google Scholar using the following terms: ‘blood stream infection’, ‘mechanical ventilation’, and ‘intensive care unit’, each combined with either ‘meta-analysis’ or ‘systematic review’ from 1985 up to October 2025. Cochrane reviews and other systematic reviews were used to assist in locating additional studies together with additional studies being found by snowball sampling from the literature using the ‘Related articles’ function within Google Scholar.

The inclusion criteria were cohorts of ICU patients requiring a prolonged ICU stay which list *Candida* species among the BSI isolates. Data were extracted, where possible, for each identifiable sub-cohort representing different age groups, patient types, or observation eras from the studies. Studies limited to pediatric cohorts were not excluded.

The following studies were excluded: duplicate studies, studies with a mean length of stay (LOS) < 3 days, studies with < 30 patients and studies with data for less than three bacterial BSI types in addition to data for candidemia. Non-concurrent control groups within studies of antibiotic-based decontamination interventions used to prevent ICU-acquired infections were included, but concurrent control groups were excluded, as these potentially have an altered correlation between the five bacteria and *Candida* species as a result of rebound and spillover.

### Outcomes of interest

The independent variable in the regression models was the count of patients with *Candida* species listed among the BSI isolates as a proportion of the total number of patients in each cohort. The dependent variables were the proportion of patients with BSI with each of *Pseudomonas aeruginosa, Acinetobacter* species, *Staphylococcus aureus, Enterococcal* species, and Coagulase-negative Staphylococci listed among the BSI isolates. These counts for each ICU cohort were expressed as a proportion of the total number of patients in each cohort.

Other endpoints of interest that were extracted from the publications included the group mean or median LOS or the group mean or median duration of mechanical ventilation, the proportion of patients receiving prolonged (> 24 h) mechanical ventilation, the year of study publication, and the geographic location of the ICU. These endpoints were selected for being notable and commonly available among the studies.

The BSI isolate data were logit transformed from the original proportion data using the total number of patients in each cohort as the denominator. Note that to enable a logit transformation, the addition of 0.5 as a continuity correction is required for any zero counts to enable zero event groups to be represented on the logit scale as the logit transformation of zero is indeterminate. The LOS data are positively skewed and were log transformed for analysis.

### Data analysis

The generalized linear model (*glm)* command in Stata 18 (Stata 18, College Station, TX, USA)^14^ was used to model the relationship between BSI isolates for each of the five categories of bacterial isolates. The marginal effects associated with increasing counts of *Candida* species BSI were obtained by using the margins command following each glm model.

Given the relative rarity of some BSI associated with some bacterial species, occurring in possibly < 1% of patients among some cohorts, the analysis was initially undertaken with the inclusion of all cohorts and then repeated after exclusion of cohorts with < 100 patients as a sensitivity test.

The incidence proportion data for candidemia and each BSI incidence type were logit transformed to enable assessments of correlation and for generation of 95% prediction ellipses in regression modelling. The prediction ellipse method on a logit scale is optimal for enabling the bivariate correlation to be observed. The confidence ellipses were generated using the ‘*ellip’* command within Stata as previously.^15, 16^

## Results

The search (Supplementary Fig. 1) identified 60 cohorts derived from 50 published studies (Supplementary Tables 1, 2). The studies were published over a period of four decades (1985–2025), with the interquartile range (IQR) being 2000–2014. The median ICU-LOS was 9 days (IQR; 6–16), and the median size was 1241 patients (IQR; 361–4270). There were seven cohorts with < 100 patients. There was more than one cohort in ten publications.

Most studies originated from ICUs in North America (*n* = 19), Northern Europe (*n* = 20), or Southern Europe (*n* = 14). Four were trauma patient cohorts and, for the remaining cohorts, the median percentage of trauma patients was 14 (IQR; 0–30). There was one pediatric patient cohort arising from one study. A majority (> 50%) of patients in each cohort received prolonged mechanical ventilation, where this was stated. The proportion of the cohort who received prolonged mechanical ventilation was not stated for 13 cohorts.

The median BSI incidence proportion was 7.8% (IQR; 4.8%–11.6%). The median BSI incidence proportion per 100 patients for *Staphylococcus aureus, Pseudomonas aeruginosa, Acinetobacter* species, Enterococcal species, and Coagulase-negative Staphylococci were 1.0% (IQR; 0.5%–1.9%), 0.5% (IQR; 0.3%–1.0%), 0.13% (IQR; 0.01%–1.3%), 0.6% (IQR; 0.02%–0.9%), and 1.3% (IQR; 0.4%–2.1%), respectively. The proportion of patients with *Candida* species as a BSI isolate was 0.42% (IQR; 0.24%–0.83%).

**Figure 1:**
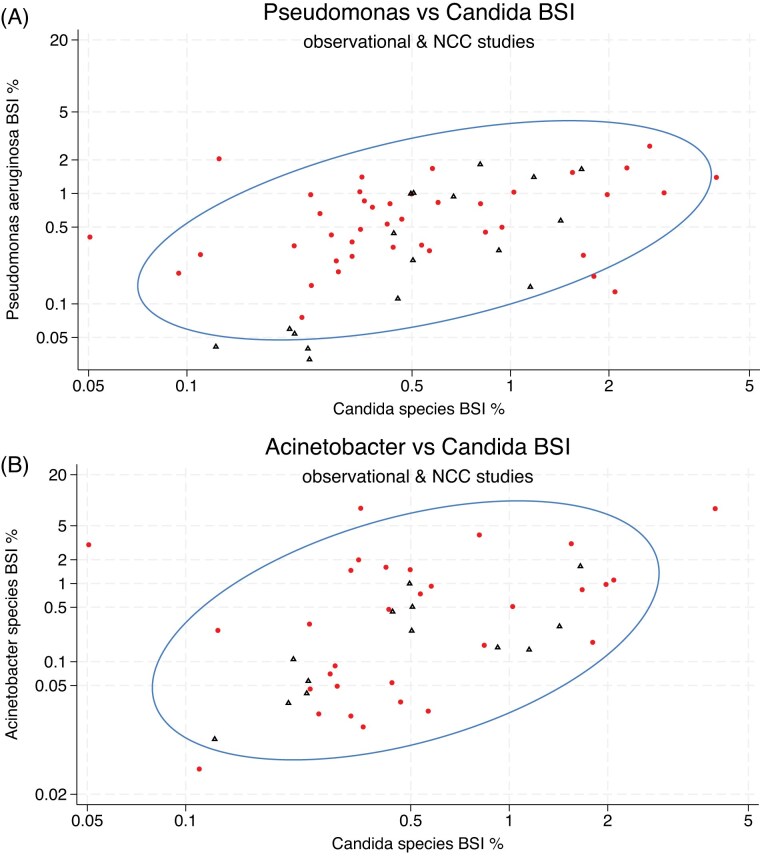
**A & B**. Correlation between the percentage of patients in each cohort with BSI with *Pseudomonas aeruginosa* versus *Candida* species among observational (red ●) & non-concurrent control groups (blue ▲) of antibiotic-based decontamination intervention studies. Figure 1A) and correlation between the percentage of patients in each cohort with BSI with *Acinetobacter* species versus *Candida* species among published studies (Fig. 1B). The ellipse is the means centered 95% confidence ellipse. Note the axes are logit scales. Figures with studies indicated are located in the supplement.

**Figure 2:**
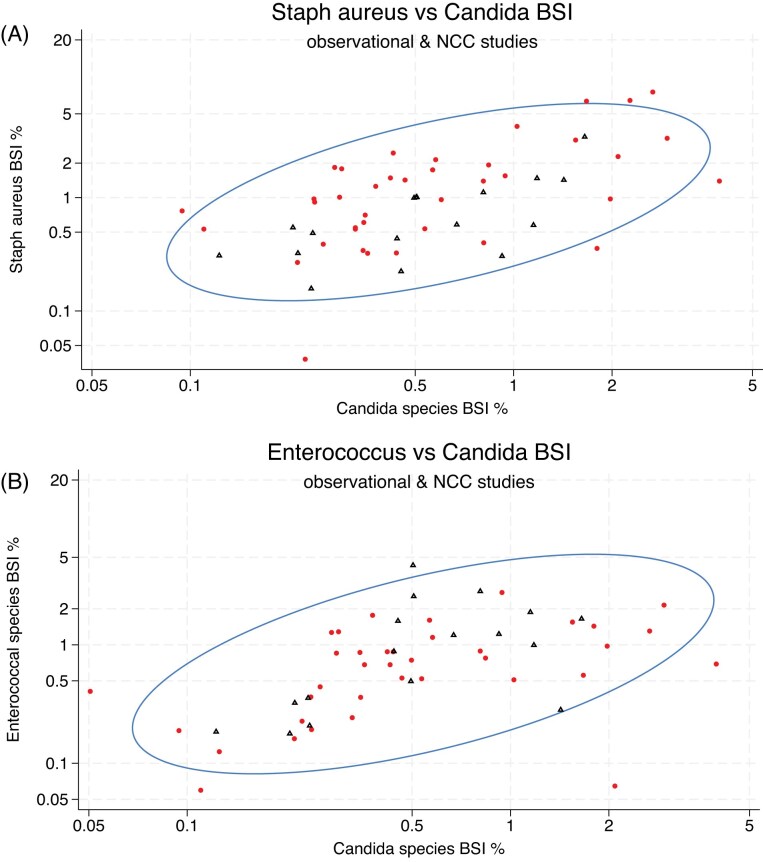
**A & B**. Correlation between the percentage of patients in each cohort with BSI with *Staphylococcus aureus* versus *Candida* species among observational (red ●) & non-concurrent control groups (blue ▲) of antibiotic-based decontamination intervention studies (Fig. 2A) and correlation between the percentage of patients in each cohort with BSI with *Enterococcal* species versus *Candida* species among published studies (Fig. 2B). The ellipse is the means centered 95% confidence ellipse. Note the axes are logit scales. Figures with studies indicated are located in the supplement.

The scatterplots of isolate counts and the accompanying ellipse plots for the BSI are presented in Figures 1, 2 and Supplementary Figures 2–6. The BSI incidence proportions with *Acinetobacter* species (correlation coefficient = 0.69), *Staphylococcus aureus* (correlation coefficient = 0.47) and *Pseudomonas aeruginosa* (0.4), but less so Coagulase-negative Staphylococci (0.37) and *Enterococcal* species (0.32), each show co-variation with the incidence proportion of Candidemia. The co-variation observed between the incidence proportion of Candidemia and the incidence proportions for each of the five bacterial BSI’s among the observational cohorts and among the Non-concurrent control groups of antibiotic-based decontamination intervention studies were similar.

### Regression models and sensitivity tests

The regression models are presented in Table 1. The *Candida* species proportion significantly co-varied with three of the five bacterial BSI proportions after adjusting for group mean ICU-LOS and year of study publication. The group mean ICU-LOS is included in all models regardless of the level of statistical significance, which, in most cases, was marginal. The results after excluding seven cohorts with < 100 patients were similar (data not shown). The results for cohorts published since the year 2000 versus before the year 2000 were similar (data not shown). In comparing studies published before versus since the year 2000, there was no evidence that the patterns of BSI isolates have changed.

**Table 1:** Regression models of isolate count data.

Model	Factor	Coefficient	95% confidence interval	*P*-value
*Pseudomonas aeruginosa*	*Candida* species^1^	0.42	+0.13 to + 0.71	.005
	ICU-LOS^2^	0.03	−0.01 to + 0.06	.093
	Year of study publication^3^	−0.04	−0.07 to -0.01	.013
	Constant	−2.39	−4.12 to -0.67	.007
*Acinetobacter* species	*Candida* species^1^	0.37	−0.22 to + 0.96	.23
	ICU-LOS^2^	0.1	0.05 to + 0.15	.001
	Year of study publication^3^	−0.03	−0.09 to + 0.03	.27
	Constant	−4.3	−7.9 to -0.73	.018
*Staphylococcus aureus*	*Candida* species^1^	0.52	+0.29 to + 0.76	.001
	ICU-LOS^2^	0.02	−0.01 to + 0.05	.061
	Year of study publication^3^	−0.03	−0.05 to -0.01	.005
	Constant	−1.41	−2.66 to -0.168	.027
Coagulase-negative Staphylococci	*Candida* species^1^	0.3	−0.04 to + 0.64	.086
	ICU-LOS^2^	0.03	−0.01 to + 0.07	.075
	Year of study publication^3^	−0.02	−0.05 to -0.02	.29
	Constant	−2.75	−4.70 to -0.8	.006
*Enterococcus* species	*Candida* species^1^	0.47	+0.22 to + 0.71	.001
	ICU-LOS^2^	0.02	−0.01 to + 0.05	.16
	Year of study publication^3^	−0.02	−0.05 to + 0.01	.06
	Constant	−2.13	−3.6 to -0.63	.005

ICU-LOS = ICU Length of stay.

1. The proportion of patients with *Candida* species among the BSI isolates.

2. Per day of ICU-LOS.

3. Per year post 1980.

An increment in *Candida* species BSI proportions from 1% to 2% (a 1 percentage point increase) corresponded to a marginal increment in each of the three bacteria of approximately 0.1–0.6 percentage points.

## Discussion

The analysis here has explored the co-variance and the degrees of association between the BSI incidence proportion with five types of bacteria and *Candida* species among ICU cohorts among published studies reporting *Candida* species and > 3 bacterial species among the BSI isolates from ICU patients.

There are three methodological considerations here in exploring these correlations between BSI isolate proportions across published cohorts. First, the literature was opportunistically searched for cohorts of ICU patients for which *Candida* species and at least three bacterial BSI isolates were reported, and the cohort denominator was known. This was undertaken to minimize reporting bias. It was important to exclude any cohorts exposed to antibiotic-based decontamination interventions, as the correlations there might have been affected, either directly or indirectly, by the effects of rebound and spillover resulting from these interventions. Second, the linear regression method used here was applied to logit-transformed data. This transformation enables the modelling of endpoints bordering on rare, with proportions typically being < 10%. Modelling using glm with a logit link was employed for this purpose. Finally, as the bacterial proportions for each bacteria together with the proportion of *Candida* species were observed concurrently in each cohort, a method using the ellipse plot was used to avoid the issue of regression to the mean for non-independent observations arising from the same cohort.

The correlations observed here are somewhat lower than the corresponding correlations found between *Candida* species and bacterial isolates from patients with ventilator-associated pneumonia among ICU patient cohorts. Note that the presence of *Candida* species among respiratory tract isolates from patients with ventilator-associated pneumonia does not necessarily imply that *Candida* species are the cause of ventilator-associated pneumonia, which is generally thought to be rare. Among ventilator-associated pneumonia isolates within 86 cohorts from 67 publications, *Staphylococcus aureus* (correlation coefficient = 0.76) and *Pseudomonas aeruginosa* (0.75), and less so *Acinetobacter* species (0.53), each show correlation with the isolation of *Candida* species. It is possible that correlations differ at different sites of infection.^17^ The BSI incidence proportions observed here correspond to summary estimates derived from a large number of studies selected without restriction to those listing *Candida* species among the BSI isolates (Table 2).^4,10,17–21^

**Table 2:** Previous estimates of proportion of BSI among ICU patient cohorts in published series

Isolate [ref]	Cohorts (*n*)	Proportion (per 100 ICU patients)	95%CI
Overall^18^	86	7.2	6.1–8.5
*Candida* species^11,18^	86	0.7	0.6–0.9
*Pseudomonas* ^10^	18	0.7	0.5–1.1
*Acinetobacter* ^17^	20	0.2	0.11–0.3
*Staphylococcus aureus* ^4,12^	29	1.5	1.1–2.0
Coagulase-negative Staphylococci^13,20^	31	1.8	1.2–2.4
Enterococci^19–21^	18	0.8	0.6–1.2

References^4,10,12,17–21^.

There is emerging concern that *Candida* species colonization might enhance colonization susceptibility among ICU patients, leading to increased invasion both from bacterial pathogens and also *Candida* species itself.^1–3^ This interaction might account for why ICU patients with *Candida* species colonization have worse outcomes, which appears not to be attributable to invasive *Candida* species infection.

*Candida* colonization is a difficult endpoint to study. *Candida* colonization is variably defined, measured and reported in the studies that have been published here. On the other hand, candidemia and bacterial BSI, which are better defined, might serve as proxy indicators of colonization among the ICU patient cohorts.

This potential interaction underlies novel infection prevention interventions that modify the ICU patient microbiome using anti-fungal agents.^5–8^ Various interventional approaches have been used in the clinical studies attempting to better define its clinical significance. For example, fluconazole prophylaxis was associated with a decreased rate of Coagulase-negative Staphylococcal infections among pre-term neonates,^7^ although this was not observed in a subsequent randomized trial.^8^

Fungi and bacteria can interact in at least four ways.^1–4,22–24^ By directly binding bacteria, *Candida* species might potentially facilitate intestinal translocation into the host. Secondly, the release or consumption of chemical compounds, such as metabolic byproducts or quorum-sensing molecules, in confined environments might enable communication between bacteria and fungi. Thirdly, biochemical changes, through either the consumption of oxygen or alterations in pH resulting from proton release, might be a mechanism. Finally, the host immune response, in both specific and nonspecific immune system responses, may be altered by the presence of *Candida* species. These interactions of bacteria within the microbiome might mediate effects that could be either synergistic or antagonistic.

For example, an inoculum of *P. aeruginosa* in the respiratory tract, which alone is insufficient to cause bacterial pneumonia within a rodent pneumonia model, is able to induce infection when co-instilled with *Candida albicans*.^23^ This enhancement was not seen where ethanol-killed *C. albicans* was co-instilled, indicating that the enhancement requires viable *Candida* species cells to be able to affect the virulence of *P. aeruginosa*. This enhancement has also been observed across challenge infections with other bacterial pathogens, including *Escherichia coli* and *Staphylococcus aureus*.

Whilst the manipulation of microbial components within the microbiome is straight forward within animal models, clinical studies are primarily correlative as these types of manipulations are not simple to study in humans.

There are several technical challenges to studying the significance of any interactions between fungi and bacteria. Moreover, *Candida* species colonization may have effects beyond its mere presence within the colonizing flora. Many of the interaction effects mediated by *Candida* species on bacteria defined in experimental settings relate to the functional activity of the *Candida* species. It is not possible to gauge this functional activity within patients as clinical reports usually list only the mere presence or absence among the colonizing flora. Moreover, the density and functional activity of the colonizing *Candida* species are not apparent from the simple listing of *Candida* species presence within the colonizing flora.

The colonization risk factors associated with the organisms under study here show some commonality. For example, the population level of use of antimicrobials within the ICU is a major risk factor for acquiring colonization with each of *Staphylococcus aureus, Enterococcal* species, Gram-negative bacteria, *Clostridium difficile*, and *Candida* species.^25,26^ Other risk factors that are difficult to measure are the risk of cross-infection, staffing factors, and the colonization density of the various microbes within the ICU being the number of patients that are colonized.^27–31^

The studies included here largely predated the emergence of *C. auris* as a pathogen of concern.

## Limitations

The first limitation is that the ICU population studies here are heterogeneous, having been published over four decades and broadly selected. This heterogeneity may also be a strength in that the findings are broadly representative of the literature experience rather than being specific to any single population. The year of study publication only approximates the actual period of study. This is a minor concern given the four-decade time period over which the studies were conducted. Also, the year of study publication was not a significant factor in any of the regression models.

Second, individual patient data would have provided greater precision in the analysis. This would allow greater certainty on the relationship and the timing of the BSI occurring within these cohorts. However, this was not available for studies that were mostly published several decades ago. The source of BSI has not been considered here and was rarely stated in the primary studies.

Third, being a group-level analysis, the inferences from the regression models relate to population-level inferences rather than inferences at the patient level. Drawing inferences from patient-level associations from group-level associations incurs the risk of ecological bias. Of note, for a range of pathogens, LOS in the ICU is strongly correlated with colonization. Hence, estimates derived here using group-level mean LOS estimates would likely underestimate the strength of LOS as a patient-level factor towards correlates of colonization. Moreover, the possibility of more complex non-linear relationships with the emergence of different patient infections in various ICU populations is not conveyed by the use of group mean ICU-LOS as a linear term within the models. For example, head injury and various forms of trauma are known to increase the risk of *Acinetobacter* species as an important patient isolate. However, this association may be confounded by the LOS among the trauma patients in the ICU setting.^32^

Fourth, the number of cohorts here was limited and potentially underpowered. Specifically, this is a limitation of the method towards demonstrating regional and temporal trends that were previously apparent when derived from analyses with larger number of more broadly selected studies.^17–21^ Excluding studies with < 100 patients did not change the findings.

Finally, colonization has not been directly studied here. There are several impediments to defining the possible clinical relevance of *Candida* species colonization within the ICU patient microbiome. Being variably defined, measured and reported, *Candida* species colonization is a difficult clinical end-point to study. Colonization is defined differently in different studies depending on the methods, sites and timings of its assessment. By using the listing among the BSI isolates of each of *Candida* species and the potentially associated bacteria enables comparability across the studies included here. Moreover, BSI are difficult to study due to the relative rarity of these endpoints. This is especially relevant for the smaller studies in which the sample size would have been a limiting factor towards observing these rare outcomes.

In conclusion, there is increasing interest in the impact of fungal dysbiosis on human health, including methods to address colonization by problematic microbes, including fungi that colonize the ICU patient microbiome together with other associations.^33, 34^

Among 60 cohorts from 50 publications, the BSI incidence proportion with five types of bacteria shows various degrees of co-variance with the candidemia incidence proportion among ICU patient cohorts. The closest association was between BSI with *Candida* species and *Acinetobacter* species. The associations estimated here contribute to our understanding of microbial interactions occurring in ICU patient populations and serve as a basis for future interventional studies that target fungal colonization towards mitigating the risk of various BSI’s.

## Supplementary Material

myag023_Supplemental_File

## Data Availability

The data analyzed during the current study are provided in Table 1.
