# Supplementary material for: Co-variation between blood stream infections with Candida species versus Pseudomonas aeruginosa, Staphylococcus aureus, and other isolates among 60 ICU patient cohorts
Source: Med Mycol. 2026 Mar 19;64(3):myag023. doi: 10.1093/mmy/myag023 (PMC13037462; doi:10.1093/mmy/myag023)
Supplement: myag023_Supplemental_File [file myag023_supplemental_file.docx]

Additional file contents:

Table s1: Observational studies 2 - 3

Table s2: Studies of ABD interventions with non-concurrent control (NCC) groups 3

References S1 – S50 4–6

Fig s1: Study selection and cohort decant 8

Fig s2: Pseudomonas correlation with Candida BSI 9

Fig s3: Acinetobacter correlation with Candida BSI 10

Fig s4: Staph aureus correlation with Candida BSI 11

Fig s5: Enterococcus correlation with Candida BSI 12

Fig s6: CNS correlation with Candida BSI 13

Table abbreviations

LOS = Length of ICU stay

MVP = mechanical ventilation (> 24 hours) proportion

Overall BSI = count of patients with blood stream infections

Can = Count of Candida

Ps = count of Pseudomonas

Ac = Count of Acinetobacter

Sr = Count of Staphylococcus aureus

CNS = Count of Coagulase negative Staphylococcus

Ent = Count of Enterococcus

ABD = antimicrobial based decontamination

CC = concurrent control;

NCC = non- concurrent control

**Table s1: Observational studies**

| Author | Year | Ref | LOS | MVP | Patients  (n) | overall BSI | Can | Ps | Ac | Sr | CNS | Ent |
| --- | --- | --- | --- | --- | --- | --- | --- | --- | --- | --- | --- | --- |
| Adrie | 2017 | 1 | 8 | 58 | 10041 | 571 | 60 | 83 |  | 96 |  |  |
| Baldesi | 2017 | 2 | 7 | 64 | 246459 | 9087 | 851 | 1172 |  | 1490 | 2163 | 891 |
| Cade | 1993 | 3 | 16 | 98 | 98 | 8 | 1 | 1 | 0 | 4 | 1 | 0 |
| Craven-medical | 1988 | 4 | 6 | 100 | 526 | 25 | 0 | 1 |  | 4 | 0 | 1 |
| Craven-surgical | 1988 | 4 | 6 | 100 | 799 | 60 | 3 | 6 |  | 10 | 11 | 14 |
| de_Santis | 2000 | 5 |  |  | 713 | 60 | 2 | 3 |  | 13 | 17 | 9 |
| de_Santis | 2013 | 5 |  |  | 1318 | 37 | 3 | 1 |  | 0 | 16 | 3 |
| Edgeworth '71-'85 | 1985 | 6 |  |  | 11721 | 241 | 13 | 33 | 0 | 62 | 21 | 7 |
| Edgeworth '86-'95 | 1995 | 6 | 5 |  | 9272 | 329 | 24 | 61 | 2 | 36 | 40 | 41 |
| El-Masri | 2004 | 7 | 11 |  | 361 | 55 | 6 | 1 | 3 | 24 | 6 | 2 |
| Esteve | 2007 | 8 | 17 | 78 | 404 | 47 | 2 | 4 | 6 |  | 15 | 3 |
| Esteve | 2007 | 8 | 16 | 80 | 395 | 43 | 0 | 8 | 1 |  | 23 | 0 |
| Garcı´a-Garmendia | 2001 | 9 | 5 | 46 | 2640 | 233 | 11 | 14 | 42 | 39 | 46 | 23 |
| Garrouste-Orgeas | 2006 | 10 | 11 | 75 | 3247 | 232 | 15 | 19 | 1 | 46 | 50 | 17 |
| Giamarellos-Bourboulis | 2009 | 11 | 12 | 100 | 72 | 18 | 3 | 1 | 2 | 1 | 5 | 0 |
| Gouel-Cheron_all | 2022 | 12 | 4 | 15 | 143589 | 6906 | 350 | 212 | 65 | 1309 | 582 | 277 |
| Holzapfel_99 | 1999 | 13 | 15 | 100 | 200 | 46 | 1 | 0 | 0 | 2 | 29 | 9 |
| Holzapfel_99 | 1999 | 13 | 17 | 100 | 199 | 36 | 1 | 2 | 1 | 2 | 22 | 5 |
| Ibrahim'00 | 2000 | 14 | 11 | 69 | 4913 | 492 | 41 | 22 | 8 | 94 | 96 | 38 |
| Kallel | 2020 | 15 | 26 | 93 | 2353 | 223 | 10 | 19 | 11 | 57 | 25 | 16 |
| Kollef '97_C | 1997 | 16 | 4 | 90 | 353 | 13 | 5 | 2 | 1 | 5 | 0 | 1 |
| Kollef '97_post | 1997 | 16 | 4 | 90 | 327 | 11 | 3 | 1 | 0 | 1 | 0 | 4 |
| Kolpa | 2018 | 17 | 19 | 100 | 1847 | 156 | 6 | 5 | 27 | 10 | 69 |  |
| Laggner | 1989 | 18 | 32 |  | 32 | 4 | 0 | 0 | 1 | 1 |  | 0 |
| Laupland | 2002 | 19 | 5 | 84 | 1017 | 51 | 3 | 2 | 0 | 18 | 9 | 13 |
| Laupland | 2004 | 20 | 5 |  | 4473 | 189 | 13 | 11 | 4 | 45 | 28 | 38 |
| Lim | 2014 | 21 | 41 | 100 | 1545 | 129 | 32 | 2 | 17 | 35 | 7 | 1 |
| Magnason | 2008 | 22 | 8 | 70 | 280 | 25 | 5 | 0 | 0 | 1 | 9 | 4 |
| Massart | 2021 | 23 | 6 | 84 | 2464 | 71 | 8 | 9 | 0 | 13 | 7 | 6 |
| Mitsogianni | 2010 | 24 | 16 |  | 143 | 35 | 0 | 2 | 12 | 1 | 1 |  |
| Mitsogianni | 2011 | 24 | 16 |  | 124 | 18 | 1 | 1 | 5 | 0 | 0 |  |
| Montecalvo_J | 1992 | 25 | 10 | 100 | 38 | 10 | 1 | 1 |  | 3 | 2 | 0 |
| Orsi | 2007 | 26 | 36 | 98 | 1741 | 167 | 10 | 29 | 16 | 37 | 61 | 20 |
| Orsi | 2012 | 26 | 34 | 100 | 1165 | 83 | 4 | 12 | 23 | 4 | 17 | 10 |
| Osmon | 2003 | 27 | 8 | 72 | 893 | 118 | 26 | 9 |  | 29 | 17 | 19 |
| Prowle | 2011 | 28 | 6 | 69 | 6339 | 330 | 51 |  |  | 88 | 80 | 56 |
| Rello'94 | 1994 | 29 | 7 | 72 | 1650 | 111 | 4 | 16 | 5 | 16 | 21 | 6 |
| Roimi_BIDMC | 2020 | 30 | 9 | 78 | 7419 | 151 | 17 | 9 | 1 | 27 |  | 49 |
| Roimi_RHCC | 2020 | 30 | 15 | 67 | 1812 | 162 | 16 | 25 | 26 | 20 |  | 12 |
| Rozaidi | 2001 | 31 | 5 | 65 | 988 | 80 | 0 | 4 | 30 |  | 16 | 4 |
| Schlechte | 2023 | 32 | 7 | 100 | 51 | 3 | 1 | 0 | 0 | 0 | 2 | 0 |

**Table s1 (continued): Observational studies**

| Author | Year | Ref | LOS | MVP | Patients  (n) | overall BSI | Can | Ps | Ac | Sr | CNS | Ent |
| --- | --- | --- | --- | --- | --- | --- | --- | --- | --- | --- | --- | --- |
| Stolcin | 2020 | 33 | 6 | 27 | 3388 | 213 | 12 | 29 | 0 | 11 | 11 | 23 |
| Thompson | 2008 | 34 | 6 |  | 4270 | 210 | 24 | 13 | 1 | 74 | 89 | 68 |
| Urli | 2002 | 35 | 21 | 95 | 178 | 40 | 4 | 3 |  | 12 | 11 |  |
| Warren | 2001 | 36 | 4 | 28 | 920 | 34 | 4 | 3 | 0 | 3 | 18 | 8 |
| Xie | 2020 | 37 | 25 | 67 | 8474 | 486 | 45 | 29 | 62 | 45 | 42 | 44 |

Table s1 footnotes

**Initial data sources**

Safdar N, Dezfulian C, Collard HR, Saint S. Clinical and economic consequences of ventilator-associated pneumonia: a systematic review. Crit Care Med 2005;33(10):2184-93.

Melsen WG, Rovers MM, Bonten MJM. Ventilator-associated pneumonia and mortality: A systematic review of observational studies. Crit Care Med 2009; 37:2709–18.

Agrafiotis M, Siempos II, Ntaidou TK, Falagas ME. Attributable mortality of ventilator-associated pneumonia: a meta-analysis. Internat J Tuberculosis Lung Dis. 2011;15(9):1154-63.

Pileggi C, Mascaro V, Bianco A, Nobile CG, Pavia M. Ventilator bundle and its effects on mortality among ICU patients: a meta-analysis. Crit Care Med. 2018;46(7):1167-74.

**Table s2: ABD intervention studies with non-concurrent control groups ^a^**

| Author | Year | Ref | LOS | MVP | Patients  (n) | overall BSI | Can | Ps | Ac | Sr | CNS | Ent |
| --- | --- | --- | --- | --- | --- | --- | --- | --- | --- | --- | --- | --- |
| Camus '14 _C | 2014 | 38 | 4 | 28 | 925 | 36 | 2 | 0 | 1 | 3 | 7 | 3 |
| de Smet _C | 2009 | 39 | 9 | 88 | 1990 | 185 | 16 | 36 |  | 22 |  | 55 |
| Hartenauer _C | 1990 | 40 | 14 | 100 | 101 | 7 | 0 | 1 | 1 | 1 | 1 | 0 |
| Massart_ECMO_C | 2023 | 41 | 22 | 100 | 61 |  | 8 | 5 |  | 1 | 4 | 11 |
| Massart_REAREZO_C | 2025 | 42 | 10 | 100 | 2727 | 261 | 32 | 38 |  | 40 | 42 | 27 |
| Wittekamp _C | 2018 | 43 | 10 | 100 | 2251 | 154 | 15 | 21 |  | 13 | 97 | 27 |
| Fourrier'00 | 2000 | 44 | 24 | 100 | 30 | 3 | 0 | 0 | 0 | 1 | 1 | 0 |
| Fourrier'05 | 2005 | 45 | 13 | 100 | 114 | 3 | 0 | 0 | 0 | 0 | 2 | 1 |
| Bleasdale | 2007 | 46 | 5 | 35 | 445 | 27 | 2 | 0 |  | 1 | 15 | 7 |
| Climo_CRT | 2013 | 47 | 6 |  | 1398 | 127 | 16 | 2 | 2 | 8 | 34 | 26 |
| Milstone | 2013 | 48 | 3 |  | 2525 | 79 | 6 | 1 | 1 | 4 | 38 | 9 |
| Noto_pre | 2015 | 49 | 3 |  | 4852 | 95 | 6 | 2 | 0 | 15 | 37 | 9 |
| Huang | 2013 | 50 | NR |  | 15816 | 265 | 38 | 5 | 9 | 77 | 48 | 33 |
| Huang | 2013 | 50 | NR |  | 23480 | 360 | 49 | 14 | 7 | 128 | 54 | 42 |

References

1. Adrie C, Garrouste-Orgeas M, Essaied WI, Schwebel C, Darmon M, Mourvillier B, Ruckly S, Dumenil AS, Kallel H, Argaud L, Marcotte G. Attributable mortality of ICU-acquired bloodstream infections: impact of the source, causative micro-organism, resistance profile and antimicrobial therapy. *J Infection*. 2017 ;74(2):131-41.
2. Baldesi O, Bailly S, Ruckly S, Lepape A, L'Heriteau F, et al. ICU-acquired candidaemia in France: epidemiology and temporal trends, 2004–2013–a study from the REA-RAISIN network. *J Infect.* 2017;75(1):59-67.
3. Cade JF, McOwat E, Siganporia R, Keighley C, Presneill J, Sinickas V: Uncertain relevance of gastric colonization in the seriously ill. *Intensive Care Med*. 1992;18:210-217
4. Craven DE, Kunches LM, Lichtenberg DA, Kollisch NR, Barry MA, Heeren TC, McCabe WR: Nosocomial infection and fatality in medical and surgical intensive care unit patients. *Arch Intern Med.* 1988;148:1161-1168
5. de Santis V, Gresoiu M, Corona A, Wilson AP, Singer M. Bacteraemia incidence, causative organisms and resistance patterns, antibiotic strategies and outcomes in a single university hospital ICU: continuing improvement between 2000 and 2013. J Antimicrob Chemoth. 2015;70(1):273-8.
6. Edgeworth JD, Treacher DF, Eykyn SJ. A 25-year study of nosocomial bacteremia in an adult intensive care unit. *Crit Care Med*. 1999 ;27(8):1421-8.
7. El-Masri MM, Hammad TA, McLeskey SW, Joshi M, et al Predictors of nosocomial bloodstream infections among critically ill adult trauma patients. *Infect Cont & Hosp Epidemiol.* 2004;25(8):656-63.
8. Esteve F, Pujol M, Limon E, Saballs M, Argerich MJ, Verdaguer R, Manez R, Ariza X, Gudiol F. Bloodstream infection related to catheter connections: a prospective trial of two connection systems. *J Hosp Infect*. 2007;67(1):30-4.
9. García-Garmendia JL, Ortiz-Leyba C, Garnacho-Montero J, Jiménez-Jiménez FJ, et al. Risk factors for *Acinetobacter baumannii* nosocomial bacteremia in critically ill patients: a cohort study. *Clin Infect Dis*. 2001; 33(7):939-46.
10. Garrouste-Orgeas M, Timsit JF, Tafflet M, Misset B, Zahar JR, et al: Excess risk of death from intensive care unit—acquired nosocomial bloodstream infections: a reappraisal. *Clin Infect Dis* 2006, 42:1118-1126.
11. Giamarellos-Bourboulis EJ, Bengmark S, Kanellakopoulou K, et al. Pro-and synbiotics to control inflammation and infection in patients with multiple injuries. *J Trauma & Acute Care Surg*. 2009; 67:815-21
12. Gouel-Cheron A, Swihart BJ, Warner S, Mathew L, Strich JR, Mancera A, Follmann D, Kadri SS. Epidemiology of ICU-Onset Bloodstream Infection: Prevalence, Pathogens, and Risk Factors Among 150,948 ICU Patients at 85 US Hospitals. *Crit Care Med*. 2022;50(12):1725-36.
13. Holzapfel L, Chastang C, Demingeon G, Bohe J, Piralla B, Coupry A: A randomized study assessing the systematic search for maxillary sinusitis in nasotracheally mechanically ventilated patients. Influence of nosocomial maxillary sinusitis on the occurrence of ventilator-associated pneumonia. *Am J Respir Crit Care Med*. 1999;159:695-701
14. Ibrahim EH, Sherman G, Ward S, Fraser VJ, Kollef MH. The influence of inadequate antimicrobial treatment of bloodstream infections on patient outcomes in the ICU setting. *Chest*. 2000;118(1):146-55.
15. Kallel H, Houcke S, Resiere D, Roy M, Mayence C, Mathien C, Mootien J, Demar M, Hommel D, Djossou F. Epidemiology and Prognosis of Intensive Care Unit–Acquired Bloodstream Infection. *Am J Trop med & Hygiene.* 2020;103(1):508.
16. Kollef MH, Vlasnik JO, Sharpless L, Pasque C, Murphy D, Fraser V. Scheduled change of antibiotic classes: a strategy to decrease the incidence of ventilator-associated pneumonia. *Am J Respir Crit Care Med*. 1997;156(4):1040-8.
17. Kołpa M, Wałaszek M, Gniadek A, Wolak Z, Dobroś W. Incidence, microbiological profile and risk factors of healthcare-associated infections in intensive care units: a 10 year observation in a provincial hospital in Southern Poland. *International J environ res & public health*. 2018;15(1):112.
18. Laggner AN, Lenz K, Base W, Druml W, Schneeweiss B, Grimm G: Prevention of upper gastrointestinal bleeding in long-term ventilated patients. Sucralfate versus ranitidine. Am J Med. 1989;86:81-4.
19. Laupland KB, Zygun DA, Davies HD, Church DL, Louie TJ, Doig CJ Population-based assessment of intensive care unit-acquired bloodstream infections in adults: incidence, risk factors, and associated mortality rate. *Crit Care Med* 2002;30:2462-2467.
20. Laupland KB, Kirkpatrick AW, Church DL, Ross T, Gregson DB Intensive-care-unit-acquired bloodstream infections in a regional critically ill population. *J Hosp Infect* 2004;58(2): 137-145.
21. Lim SJ, Choi JY, Lee SJ, Cho YJ, Jeong YY, Kim HC, Lee JD, Hwang YS. Intensive care unit-acquired blood stream infections: a 5-year retrospective analysis of a single tertiary care hospital in Korea. Infection. 2014;42:875-81.
22. Magnason S, Kristinsson KG, Stefansson T, Erlendsdottir H, Jonsdottir K, Kristjansson M, et al: Risk factors and outcome in ICU‐acquired infections. *Acta Anaesthesiologica Scandinavica*. 2008;52:1238-1245
23. Massart N, Wattecamps G, Moriconi M, Fillatre P. Attributable mortality of ICU acquired bloodstream infections: a propensity-score matched analysis. *Eur J Clin Microbiol & Infect Dis.* 2021;40(8):1673-80.
24. Mitsogianni M, Vasileiadis I, Parisi M, Tzanis G, Kampisiouli E, Psaroudaki Z, Perivolioti E, Fountoulis K, Routsi C, Nanas S, Tsiodras S. A Multifaceted Intervention Program to Prevent Bloodstream Infection in an Intensive Care Unit. *Health Science J*. 2016;10(2):1.
25. Montecalvo MA, Steger KA, Farber HW, Smith BF, Dennis RC, Fitzpatrick GF, Pollack SD, Korsberg TZ, Birkett DH, Hirsch EF. Nutritional outcome and pneumonia in critical care patients randomized to gastric versus jejunal tube feedings. The Critical Care Research Team. *Crit Care Med*. 1992 ;20(10):1377-87.
26. Orsi GB, Giuliano S, Franchi C, Ciorba V, Protano C, Giordano A, Rocco M, Venditti M. Changed epidemiology of ICU acquired bloodstream infections over 12 years in an Italian teaching hospital. *Minerva Anestesiol.* 2015;81(9):980-8.
27. Osmon S, Warren D, Seiler SM, Shannon W, Fraser VJ, Kollef MH: The influence of infection on hospital mortality for patients requiring >48 h of intensive care. *Chest* 2003, 124:1021-1029.
28. Prowle JR, Echeverri JE, Ligabo EV, Sherry N, Taori GC, Crozier TM, Bellomo R. Acquired bloodstream infection in the intensive care unit: incidence and attributable mortality. *Crit Care* 2011;15(2):R100.
29. Rello J, Ricart M, Mirelis B, Quintana E, Gurgui M, Net A, Prats, G: Nosocomial bacteremia in a medical-surgical intensive care unit: epidemiologic characteristics and factors influencing mortality in 111 episodes. *Intensive Care Med* 1994;20:94-98.
30. Roimi M, Neuberger A, Shrot A, Paul M, Geffen Y, Bar-Lavie Y. Early diagnosis of bloodstream infections in the intensive care unit using machine-learning algorithms. *Intensive Care Med*. 2020;46:454-62.
31. Rozaidi SW, Sukro J, Dan A. The incidence of nosocomial infection in the Intensive Care Unit, Hospital Universiti Kebangsaan Malaysia: ICU-acquired nosocomial infection surveillance program 1998-1999. *The Medical Journal of Malaysia.* 2001; 56(2):207-22.
32. Schlechte J, Zucoloto AZ, Yu IL, Doig CJ, Dunbar MJ, McCoy KD, McDonald B. Dysbiosis of a microbiota–immune metasystem in critical illness is associated with nosocomial infections. *Nature Medicine*. 2023;29(4):1017-27.
33. Stoclin A, Rotolo F, Hicheri Y, Mons M, Chachaty E, Gachot B, Pignon JP, Wartelle M, Blot F. Ventilator-associated pneumonia and bloodstream infections in intensive care unit cancer patients: a retrospective 12-year study on 3388 prospectively monitored patients. *Supportive Care in Cancer*. 2020;28:193-200.
34. Thompson DS. Estimates of the rate of acquisition of bacteraemia and associated excess mortality in a general intensive care unit: a 10 year study. *J Hosp Infect*. 2008;69(1):56-61.
35. Urli T, Perone G, Acquarolo A, Zappa S, Antonini B, Ciani A: Surveillance of infections acquired in intensive care: usefulness in clinical practice. *J Hosp Infect* 2002, 52:130-5.
36. Warren DK, Zack JE, Elward AM, Cox MJ, Fraser VJ. Nosocomial primary bloodstream infections in intensive care unit patients in a nonteaching community medical center: a 21-month prospective study. *Clin Infect Dis.* 2001;33(8):1329-35.
37. Xie DS, Xiong W, Lai RP, Liu L, Gan XM, Wang XH, Wang M, Lou YX, Fu XY, Wang HF, Xiang H. Ventilator-associated pneumonia in intensive care units in Hubei Province, China: a multicentre prospective cohort survey. *J Hosp Infect.* 2011;78(4):284-8.
38. Camus C, Salomon S, Bouchigny C, Gacouin A, Lavoué S, Donnio PY, Bellissant E (2014) Short-Term Decline in All-Cause Acquired Infections With the Routine Use of a Decontamination Regimen Combining Topical Polymyxin, Tobramycin, and Amphotericin B With Mupirocin and Chlorhexidine in the ICU: A Single-Center Experience. *Crit Care Med* 42:1121-1130
39. de Smet AMGA, Kluytmans JAJW, Cooper BS, et al: Decontamination of the digestive tract and oropharynx in ICU patients. *N Engl J Med* 2009, 360:20–31.
40. Hartenauer UB, Thülig B, Lawin P, Fegeler W. Infection surveillance and selective decontamination of the digestive tract (SDD) in critically ill patients—results of a controlled study. *Infection.* 1990;18(1):S22-30.
41. Massart N, Camus C, Nesseler N, Fillâtre P, Flecher E, Mansour A, Verhoye JP, Le Fevre L, Luyt CE. Multiple-site decontamination to prevent acquired infection in patients with veno-venous ECMO support. *Annals of Intensive Care*. 2023;13(1):1-1.
42. Massart N, Leone M, Reizine F, Duclos G, Machut A, Vacheron CH, Savey A, Hammad E, Friggeri A, Lepape A. Selective decontamination regimens in French ICUs: association with reduced infection and resistance emergence. *Annals of Intensive Care*. 2025;15(1):1-9.
43. Wittekamp BH, Plantinga NL, Cooper BS, Lopez-Contreras J, Coll P, Mancebo J, Wise MP, Morgan MP, Depuydt P, Boelens J, Dugernier T. Decontamination strategies and bloodstream infections with antibiotic-resistant microorganisms in ventilated patients: a randomized clinical trial. *JAMA*. 2018 ;320(20):2087-98.
44. Fourrier FE, Cau-Pottier H, Boutigny M, Roussel-Delvallez M, Jourdain, Chopin C: Effects of dental plaque antiseptic decontamination on bacterial colonization and nosocomial infections in critically ill patients. *Intensive Care Med*. 2000;26:1239-1247
45. Fourrier F, Dubois D, Pronnier P, Herbecq P, Leroy O, Desmettre T, Roussel-Delvallez M: Effect of gingival and dental plaque antiseptic decontamination on nosocomial infections acquired in the intensive care unit a double-blind placebo-controlled multicenter study. *Crit Care Med*. 2005;33:1728-1735
46. Bleasdale SC, Trick WE, Gonzalez IM, Lyles RD, Hayden MK, Weinstein RA. Effectiveness of chlorhexidine bathing to reduce catheter-associated bloodstream infections in medical intensive care unit patients. *Arch intern med.* 2007;167(19):2073-9.
47. Climo MW, Yokoe DS, Warren DK et al. Effect of daily chlorhexidine bathing on hospital-acquired infection. *N Engl J Med* 2013; 368: 533–542.
48. Milstone AM, Elward A, Song X, Zerr DM, Orscheln R, Speck K, Obeng D, Reich NG, Coffin SE, Perl TM, Pediatric SCRUB Trial Study Group. Daily chlorhexidine bathing to reduce bacteraemia in critically ill children: a multicentre, cluster-randomised, crossover trial. *The Lancet*. 2013;381(9872):1099-106.
49. Noto MJ, Domenico HJ, Byrne DW, Talbot T, Rice TW, Bernard GR, Wheeler AP. Chlorhexidine bathing and health care–associated infections: a randomized clinical trial. *JAMA*. 2015;313(4):369-78.
50. Huang SS, Septimus E, Kleinman K, Moody J, Hickok J, Avery TR, Lankiewicz J, Gombosev A, Terpstra L, Hartford F, Hayden MK. Targeted versus universal decolonization to prevent ICU infection. *N Engl J Med*. 2013;368(24):2255-65.
51. Hurley JC. ICU-acquired candidemia within selective digestive decontamination studies: a meta-analysis. Intensive care medicine. 2015 Nov;41(11):1877-85.Soulountsi V, Schizodimos T, Kotoulas SC. Deciphering the epidemiology of invasive candidiasis in the intensive care unit: is it possible? *Infection*. 2021;49(6):1107-31.
52. Trelles M, Murillo J, Fuenmayor-González L, Yu-Liu Y, Alexander-León H, Acebo J, Cuicapuza D, Morales M, Peña C, García-Aguilera MF. Prevalence of invasive fungal infection in critically Ill patients: a systematic review and meta-analysis. *BMC Infect Dis.* 2025;25(1):896.
53. Hurley JC. Impact of selective digestive decontamination on respiratory tract Candida among patients with suspected ventilator-associated pneumonia. A meta-analysis. *Eur J Clin Microbiol Infect Dis*. **2016**, 35:1121-35.
54. Hurley JC. Structural equation modelling the relationship between anti-fungal prophylaxis and Pseudomonas bacteremia in ICU patients. *Intensive Care Med* *Experimental*. 2022 ;10(1):2.

Meta-analyses or systematic reviews with candidemia incidence proportion data [51-54]

*Figure s1.* Flow chart of study selection and subsequent decant of component groups. The three steps are as follows: (1) An electronic search for systematic reviews containing potentially eligible studies reporting candidemia incidence proportions was performed using the following search terms: “blood stream infection”, and “intensive care unit”, up to October 2025. Additional studies located outside of systematic reviews were included. (2) The identified reviews were divided into intervention studies and observational studies. (2) Duplicate studies and studies without required data were excluded. (3) Intervention studies were streamed into non-ABD intervention (to be included with observational studies and studies of ABD interventions. (4) The component groups were decanted from each study. Note: The total numbers do not equal, as some systematic reviews provide studies in more than one category, some studies provide studies in more than one category, and some studies have unequal numbers of control and intervention groups.

ABD – antimicrobial based decontamination

Exclusions:

- < 3 days mean LOS
- Duplicates
- Candidemia risk factors
- < 50 patients
- Additional data for < 2 bacteria
- Intervention and CC groups of ABD studies

Inclusion:

- ICU cohorts
- BSI data for Candida
- BSI data for bacteria

**13 studies**

**(Table s2)**

**37 studies**

**(Table s1)**

**46**

**groups**

**14**

**groups**

**2**

**Observational studies**

**Intervention studies**

**109**

**67**

**74**

**52**

Additional studies:

- Obtained by snowball sampling


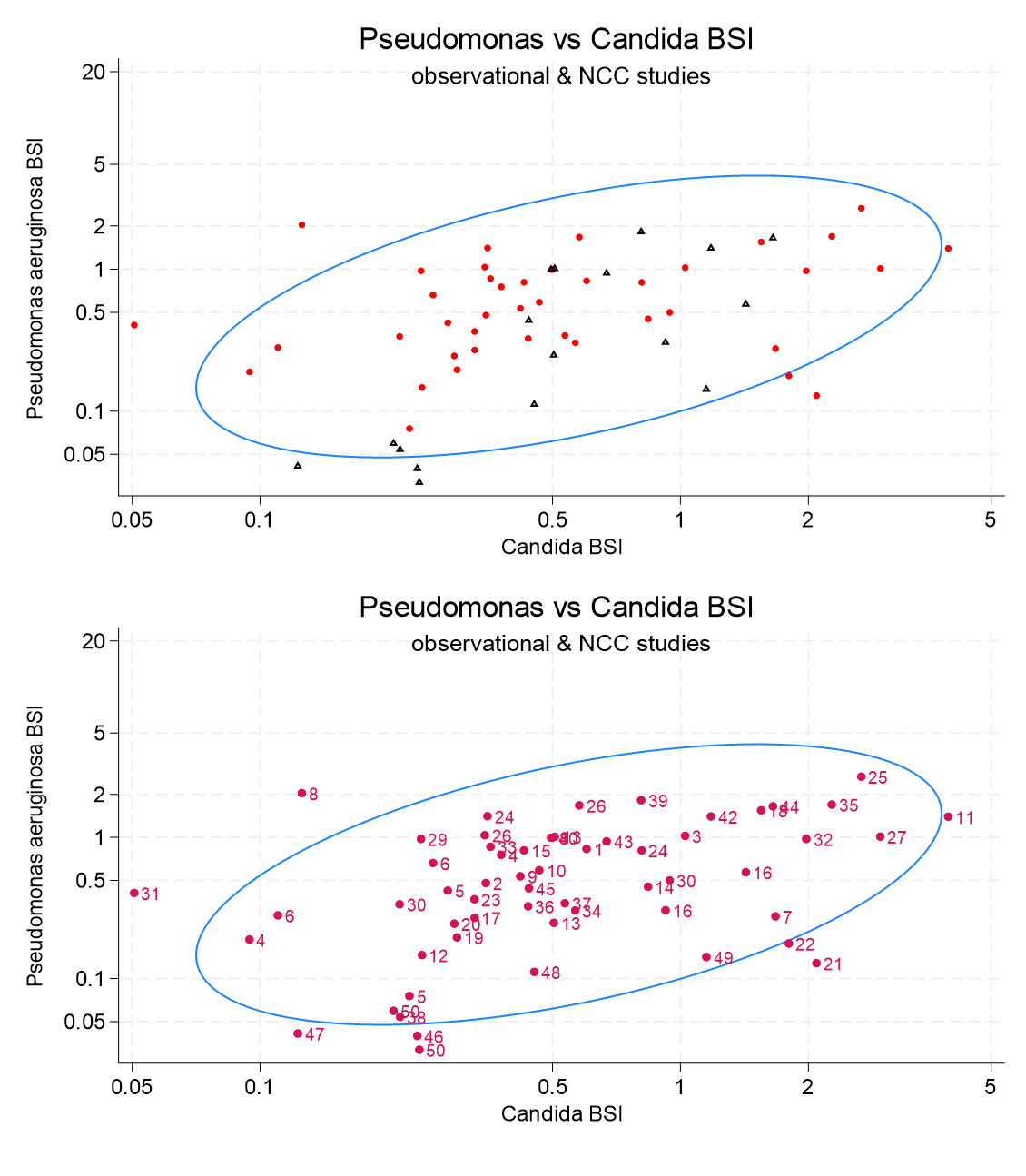


*Figure s2.* Correlation between the percentage of patients in each cohort with BSI with *Pseudomonas aeruginosa* versus Candida among published observational (red ●) & NCC groups (blue ▲) studies. The blue ellipse is the means centered 95% confidence ellipse. Note the axes are logit scales. The correlation coefficient is 0.4 (all studies). Candidemia at 0.5, 1 and 2% were associated with increments in *P aeruginosa* BSI from 0.5, 0.7 to 1.0, respectively. The bottom figure indicates the cohort ref.

*Figure s3* Correlation between the percentage of patients in each cohort with BSI with *Acinetobacter* species versus Candida among published observational (red ●) & NCC groups (blue ▲) studies. The blue ellipse is the means centered 95% confidence ellipse. Note the axes are logit scales. The correlation coefficient is 0.41. Candidemia at 0.5, 1 and 2% were associated with increments in *Acinetobacter* species BSI from 0.3, 0.4 to 0.5, respectively. The bottom figure indicates the cohort ref.

*Figure s4* Correlation between the percentage of patients in each cohort with BSI with *Staphylococcus aureus* versus Candida among published observational (red ●) & NCC groups (blue ▲) studies. The blue ellipse is the means centered 95% confidence ellipse. Note the axes are logit scales. The correlation coefficient is 0.6. Candidemia at 0.5, 1 and 2% were associated with increments in *Staphylococcus aureus* BSI from 0.9, 1.3 to 1.9, respectively. The bottom figure indicates the cohort ref.

*Figure s5* Correlation between the percentage of patients in each cohort with BSI with *Enterococcus* species versus Candida among published observational (red ●) & NCC groups (blue ▲) studies. The blue ellipse is the means centered 95% confidence ellipse. Note the axes are logit scales. The correlation coefficient is 0.56. Candidemia at 0.5, 1 and 2% were associated with increments in *Enterococcus* species BSI from 0.7, 0.9 to 1.2, respectively. The bottom figure indicates the cohort ref.

*Figure s6* Correlation between the percentage of patients in each cohort with BSI with Coagulase negative Staphylococci versus Candida among published observational (red ●) & NCC groups (blue ▲) studies. The blue ellipse is the means centered 95% confidence ellipse. Note the axes are logit scales. The correlation coefficient is 0.36. Candidemia at 0.5, 1 and 2% were associated with increments in CNS BSI from 1.2, 1.4 to 1.8, respectively. The bottom figure indicates the cohort ref.
